# Herpes Murine Model as a Biological Assay to Test Dialyzable Leukocyte Extracts Activity

**DOI:** 10.1155/2015/146305

**Published:** 2015-04-23

**Authors:** Nohemí Salinas-Jazmín, Sergio Estrada-Parra, Miguel Angel Becerril-García, Alberto Yairh Limón-Flores, Said Vázquez-Leyva, Emilio Medina-Rivero, Lenin Pavón, Marco Antonio Velasco-Velázquez, Sonia Mayra Pérez-Tapia

**Affiliations:** ^1^Unidad de Desarrollo e Investigación en Bioprocesos (UDIBI), Escuela Nacional de Ciencias Biológicas, IPN, Prolongación de Carpio y Plan de Ayala s/n, Col. Sto. Tomás, 11340 México, DF, Mexico; ^2^Departamento de Inmunología, Escuela Nacional de Ciencias Biológicas, IPN, Prolongación de Carpio y Plan de Ayala s/n, Col. Sto. Tomás, 11340 México, DF, Mexico; ^3^Instituto Nacional de Psiquiatría “Ramón De la Fuente Muñiz”, Calzada México Xochimilco 101, Col. San Lorenzo Huipulco, 14370 México, DF, Mexico; ^4^Facultad de Medicina, Universidad Nacional Autónoma de México, Ciudad Universitaria, 04510 México, DF, Mexico; ^5^Unidad de Investigación Desarrollo e Innovación Médica y Biotecnológica (UDIMEB), Escuela Nacional de Ciencias Biológicas, IPN, Prolongación de Carpio y Plan de Ayala s/n, Col. Sto. Tomás, 11340 México, DF, Mexico

## Abstract

Human dialyzable leukocyte extracts (DLEs) are heterogeneous mixtures of low-molecular-weight peptides that are released on disruption of peripheral blood leukocytes from healthy donors. DLEs improve clinical responses in infections, allergies, cancer, and immunodeficiencies. Transferon is a human DLE that has been registered as a hemoderivate by Mexican health authorities and commercialized nationally. To develop an animal model that could be used routinely as a quality control assay for Transferon, we standardized and validated a murine model of cutaneous HSV-1 infection. Using this model, we evaluated the activity of 27 Transferon batches. All batches improved the survival of HSV-1-infected mice, wherein average survival rose from 20.9% in control mice to 59.6% in Transferon-treated mice. The activity of Transferon correlated with increased serum levels of IFN-*γ* and reduced IL-6 and TNF-*α* concentrations. Our results demonstrate that (i) this mouse model of cutaneous herpes can be used to examine the activity of DLEs, such as Transferon; (ii) the assay can be used as a routine test for batch release; (iii) Transferon is produced with high homogeneity between batches; (iv) Transferon does not have direct virucidal, cytoprotective, or antireplicative effects; and (v) the protective effect of Transferon *in vivo* correlates with changes in serum cytokines.

## 1. Introduction

Governments worldwide have established standards for the production and release of biotechnological and biological drugs. For example, the US Food and Drug Administration (FDA) and Japanese Ministry of Health & Welfare (MOHW) modified their guidelines regarding biologics in 2007, and the European Medicines Agency (EMA) did so in 2008 [1–3]. In 2012, the Sanitary Risk Authority of Mexico (COFEPRIS) released the Official Mexican Standard NOM-EM-001-SSA1-2012.

A key element in the quality control of the production of biological drugs is the demonstration of their activity, and their efficacy must be preserved between commercial batches. Due to the unique nature and production of biological drugs, specific methods must be developed to evaluate their quality and attributes, including efficacy.

Human dialyzable leukocyte extracts (DLEs) are heterogeneous mixtures of low-molecular-weight peptides (<10 kDa) that are released on disruption of peripheral blood leukocytes from healthy donors [4]. DLEs are produced and commercialized worldwide. In certain countries, such as México, China, Cuba, and the Czech Republic, DLEs are registered as drugs [5–8] because they improve clinical responses in infections, allergies, cancer, and immunodeficiencies (see Berrón-Pérez et al. [9] and Viza et al. [10] for extensive reviews). Their complexity, however, has impeded an extensive characterization of their components, active substances, and biological activities.

Transferon is a human nonspecific DLE that is manufactured by the National School of Biological Sciences (ENCB), National Polytechnic Institute (IPN), Mexico, at GMP facilities. Transferon is registered by Mexican health authorities as a drug and is commercialized nationally. To establish an assay that could be used routinely as a quality control test for Transferon, we aimed to standardize and validated a method to determine its efficacy in animals. Other studies have demonstrated that the activity of DLEs can be measured by assessing the induction of delayed type hypersensitivity (DTH) in mice [11] and* in vitro* by analyzing their effects on leukocyte migration [12] or IFN-*γ* secretion [8].

DLEs are effective for treating parasitic infections (acute leishmaniasis [13] and alveolar echinococcosis [14]) and viral infections (herpes simplex virus-1 (HSV-1) and herpes zoster). Clinical trials have shown that DLEs mitigate the duration of the acute phase, the frequency of recurrences, and pain in herpes zoster patients better than acyclovir [15, 16]. These effects correlate with increased IFN-*γ* levels and CD4+ cell counts [17]. Considering the clinical effects of DLEs against herpetic infections and because animal models reflect the complexity of a drug response in an entire organism, we selected a murine model that has been reported to emulate the natural form of HSV-1 infection [18]. This murine model was standardized, validated, and used to evaluate the biological activity of 27 batches of Transferon.

## 2. Materials and Methods

### 2.1. Quality Control of Transferon

We tested Transferon batches that were produced between 2011 and 2013 using a modified version according to Borkowsky et al. [19]. Briefly, leukocytes from 1000 healthy donors were lysed with 5 freeze-thaw cycles and dialyzed against a 12-kDa membrane to obtain low-molecular-weight peptides.

The quality control of Transferon comprised (A) endotoxin content, quantified using the Endosafe-Portable Test (Charles River Laboratories, Charleston SC, USA) according to the manufacturer's instructions; the specification for endotoxin was established in Mexican Pharmacopeia, Section MGA-0316 (≤4.0 EU/mL) [20]; (B) microbiological tests, according to Mexican Pharmacopeia, Section MGA-0571 [21]; (C) physicochemical characterization by a validated ultraperformance liquid chromatography (UPLC) method [8] that analyzes molecular weights and the time of retention of the main peaks compared with those of an internal batch pattern. (D) Peptide content per final dose was measured by bicinchoninic acid (BCA) method using the Pierce BCA kit (Thermo Fisher Scientific, Waltham MA, USA) according to the manufacturer's instructions.

### 2.2. Herpes Simplex Murine Model

HSV-1 (KOS strain) was obtained from American Type Culture Collection (ATCC; Manassas VA, USA). The virus was propagated in African green monkey kidney (Vero) cells (ATCC CCL-81) that were cultured in Eagle's minimal essential medium (EMEM; ATCC) supplemented with 10% fetal bovine serum (FBS; Life Technologies, Carlsbad CA, USA). Cutaneous infection of herpes was performed by inoculating 5-week-old male BALB/c mice (Ferandelh, Mexico City, Mexico) with HSV-1, as reported [18, 22, 23]. Briefly, mice were anesthetized, and 10 *μ*L of a viral suspension that contained 5 × 10^6^ plaque-forming units (PFU)/mL was administered by cutaneous scarification on 1 cm^2^ of plucked dorsal skin [23–25]. Mice were monitored daily for 20 days to identify infection-associated symptoms, such as paralysis of the lower extremities, reduced mobility, and weight loss. When the animals experienced total loss of mobility, they were euthanized and counted as deaths for the survival analysis.

Mice had free access to food (Harlan Laboratories, Indianapolis IN, USA) and water during the experiment. All experiments were performed blindly by trained researchers.

The administration of Transferon began on day 2 after infection and continued every other day until day 10. Doses employed were 12.5 ng, 0.125 *μ*g, 0.25 *μ*g, 0.5 *μ*g, 0.75 *μ*g, 1.0 *μ*g, or 1.50 *μ*g per mouse (each weighing 14–16 g at day 0) and perorally administered in 200 *μ*L. Each experimental group comprised 10 mice. All experiments included a control group of mice that received placebo (pyrogen-free water; PISA Pharmaceutical, Mexico City, Mexico). Survival per group was plotted as Kaplan-Meier graphs and analyzed by Mantel-Cox test (*α* = 0.05) using Prism 5 Project, V 6.0 (GraphPad Software Inc, San Diego CA, USA).

#### 2.2.1. *In Vitro* Effects on HSV-1

We studied the possible direct antiviral effect of Transferon by mixing equal volumes (50 *μ*L) of Transferon (0.4 mg peptide/mL) and HSV-1 (5.17 × 10^7^ PFU7mL) and incubating the mixture for 60 minutes at 37°C. The infectivity of Transferon-treated virus was determined by analyzing the induction of a visible cytopathic effect (CPE) on Vero cells (10^4^ cells/well) at 120 h, as reported [26]. Fifty percent tissue infective dose (TCID_50_) was calculated by Spearman-Kärber method [27]. Cytopathology induced by HSV-1 (100 PFU) in presence of Transferon (10 pg/mL–10 *μ*g/mL) or acyclovir (Zoviraz, GlaxoSmithKline, Mexico City, Mexico) was determined using MTS (CellTiter 96 Aqueous One Solution cell proliferation assay reagent; Promega, Madison WI, USA) as previously reported [28, 29]. The cytoprotective effect of Transferon on target cells was evaluated by preincubating Vero cells (10^4^ cells/well) with Transferon (20 *μ*g/mL) for 24 hours before addition of HSV-1 (viral stock 5.17 × 10^7^ PFU/mL). CPE was evaluated 120 h after infection [26]; TCID_50_ calculation was performed as reported [27]. To assess the effect of Transferon on virus replication, 2 × 10^5^ Vero cells were infected with HSV-1 (2000 PFU) and incubated with Transferon (20 *μ*g/mL) or Acyclovir (10 *μ*g/mL) for 24 h at 37°C. Subsequently, viruses were recuperated from cultures through one thaw/freeze cycle and sonication. After removing cell debris by centrifugation (5000 g), the total virus yield on each well was titrated by diluting samples and incubating them with Vero cells for 120 h. We analyzed the presence of CPE at the microscope and by MTS assay. As reported by Heldt et al. [28], the TCID_50_ was defined as the concentration at which absorbance was 50% of the average absorbance from uninfected cells and was determined by nonlinear regression using Prism 6 for Mac OS X (GraphPad Software Inc).

#### 2.2.2. Cytometric Bead Array

To measure cytokine concentrations, blood samples from the orbital sinus were collected from anesthetized mice on days 7 and 9 after infection. Serum was obtained, and samples were stored at −20°C until analysis. Cytokine concentrations were determined using a cytometric bead array (CBA) mouse inflammation kit (BD Biosciences, San Diego CA, USA) according to the manufacturer's instructions. We used a FACS Aria flow cytometer and BD CBA software (BD Biosciences) for data acquisition and analysis.

#### 2.2.3. Ethics Statement

All experimental procedures with animals were performed according to the Mexican Guidelines for the Production, Care, and Use of Laboratory Animals (NOM-062-ZOO-1999) and the International Guide for the Care and Use of Laboratory Animals [30]. All efforts were made to minimize animal suffering and reduce the number of animals that were used. The experimental procedures were approved by the Ethical Committee of the Transfer Factor Project in “Escuela Nacional de Ciencias Biológicas, Instituto Politécnico Nacional” (protocol FTU/IB/012/010/PRO).

## 3. Results

### 3.1. Quality Control of Batches

All batches had endotoxin levels below 0.05 EU/mL and met the microbiological specifications in Mexican Pharmacopeia (aerobic mesophile bacteria <100 colony-forming units (CFU)/mL, filamentous fungi <10 CFU/mL, and yeasts <10 CFU/mL (Table 1)). By UPLC, we performed a physicochemical characterization of the batches. In the chromatographic profiles of the Transferon batches, we noted 8 peptidic fractions between 17 to 0.2 kDa, as previously reported [8]. The retention times of the main peaks corresponded with those of an internal pattern, defined as a batch of Transferon that satisfied all quality control tests for this product, as previously reported [8]. We also measured peptide concentrations in the Transferon samples. All batches had peptide concentrations that met the established specification of 0.400 ± 0.06 mg/mL. The average concentration in the 27 batches was 0.416 ± 0.023 mg/mL (Table 1).

### 3.2. Herpes Murine Model

**Figure 1:**
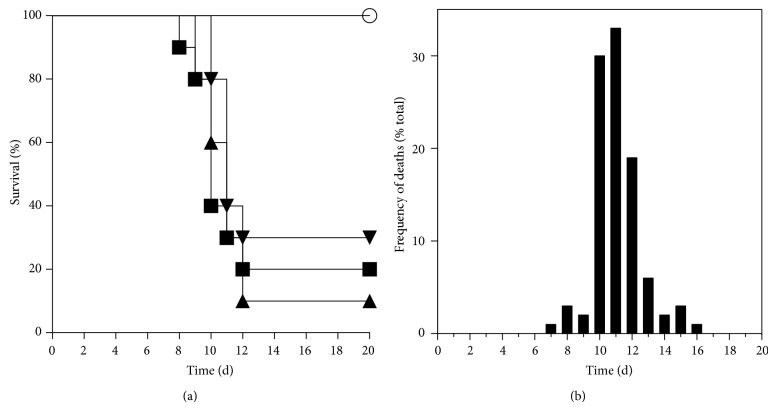
Standardization of mouse cutaneous herpes model. (a) Three different experiments performed with 5.0 × 10^4^ PFU of HSV-1 (closed symbols). In each experiment, infected mice showed significantly lower survival than controls (open circles; log-rank Mantel-Cox test; *P* < 0.001). (b) Frequency of deaths from the experiments shown in (a).

To develop a murine model of cutaneous herpes, we first studied the effect of an HSV-1 inoculum on mouse survival. We inoculated 3.5 × 10^4^, 5.0 × 10^4^, and 5.5 × 10^5^ PFU of HSV-1 and analyzed mouse survival (Figure 1). In subsequent experiments, we used 5.0 × 10^4^ PFU of HSV-1, which was the minimum inoculum that produced a decrease in survival within 70% to 100%, as reported [23–25].

Then, we determined the reproducibility of the effect of the infection by repeating the assay 3 times and measuring survival. The survival of infected animals was 10% to 30% (Figure 1(a)). Using data from the same experiments, we calculated the frequency of deaths (euthanizations) over time, noting that 85% of deaths occurred before day 13 after infection, rising to 100% by day 16 (Figure 1(b)). This information allowed us to establish an endpoint for subsequent assays.

### 3.3. Biological Evaluation of Transferon

**Figure 2:**
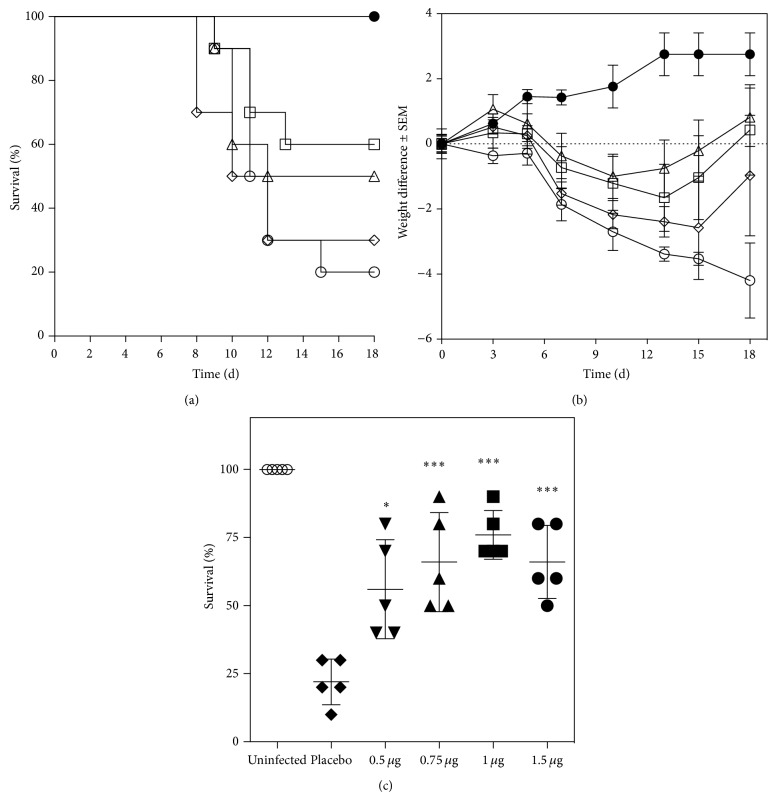
Validation of the murine herpes model for the evaluation of Transferon activity. (a) Survival curves for HSV-1 infected mice. Treatments with 1 *μ*g (open triangles) or 0.125 *μ*g (open squares) of Transferon improved survival over placebo (open circles; Log-rank Mantel-Cox test; *P* < 0.01). In contrast, 12.5 ng of Transferon (open diamonds) had no significant effect. A group of mice was left uninfected (closed circles) as control. (b) Weight changes in HSV-1 infected mice. Transferon partially protects mice from the weight loss induced by HSV-1 (symbols are as in (a)). (c) Survival induced by five different Transferon batches was evaluated during a validation protocol. All batches improved survival versus placebo (Bonferroni* t* test; ^∗^ *P* < 0.05, ^∗∗^ *P* < 0.01; ^∗∗∗^ *P* < 0.001).

To determine the doses of Transferon that were to be used to evaluate the commercial batches, we examined a ≈100-fold range of doses (12.5 ng–1.50 *μ*g per mouse). Doses above 0.125 *μ*g/mouse were equally efficacious in reducing HSV-1-induced mortality and weight loss. In contrast, 12.5 ng was ineffective, indicating that the activity of Transferon is dose-dependent. As expected, uninfected mice gained weight and showed 100% survival (Figures 2(a) and 2(b)).

The model was validated using 5 batches that were produced in 2011 and tested at 4 doses (Figure 2(c)). We evaluated (i) system specificity, by analyzing responses in placebo- and Transferon-treated animals; the average survival in controls was 23.3% (range 10% to 30%), whereas all Transferon doses induced a significant increase in survival (average increase versus placebo (Δ survival) 43.5%); (ii) system precision, by calculating the relative standard deviation (RSD) percentage for the results with each Transferon dose; % RSD was below 30% in all cases (range: 11.7% to 28.3%); (iii) system suitability, by corroborating that at least 1 dose produced a Δ survival ≥ 40%. All parameters were within the limits that were established in the validation protocol.

**Figure 3:**
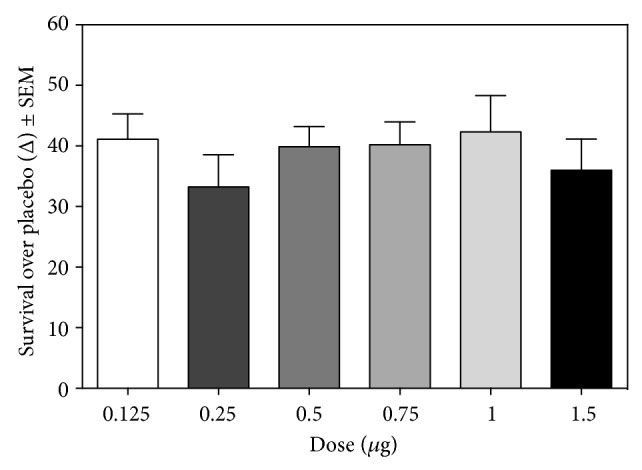
Evaluation of biological activity of 27 Transferon batches in the validated murine model of herpes. Statistical analysis (ANOVA, Bonferroni's Multiple Comparison Test) showed no differences between doses.

Eighteen batches that were produced in 2012 and 9 that were generated in 2013 were examined in the validated murine HSV-1 model. In certain batches, we evaluated 2 additional doses (0.125 and 0.25 *μ*g per mouse). All batches improved the survival of HSV-1-infected mice by an average of 37.8% (range: 30.5% to 43.3%) (Figure 3). There were no differences in average survival between doses, indicating that any of these doses could be used in future quality control assays.

### 3.4. Effects of Transferon on HSV-1 Infectivity

**Figure 4:**
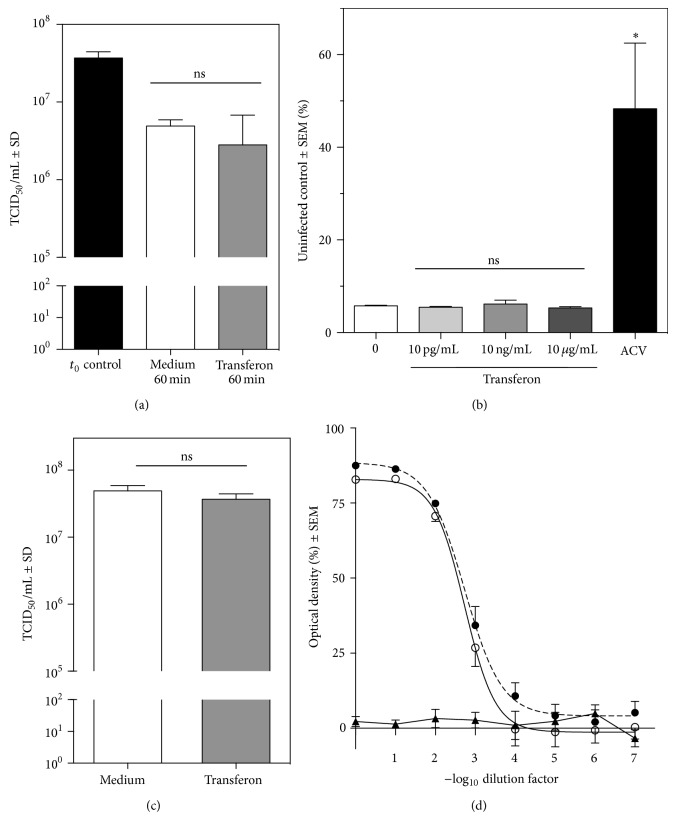
*In vitro* evaluation of antiviral activity of Transferon. (a) HSV-1 was preincubated for 60 min with Transferon (20 *μ*g/mL) or medium before evaluation of visible cytopathic effect (CPE) on Vero cells. Viruses with no preincubation (*t*_0_) were used as control (ANOVA, Bonferroni's Multiple Comparison Test; ns: nonsignificant). (b) Effect of Transferon (10 pg/mL–10 *μ*g/mL) on HSV-1-induced cytopathology, evaluated by MTS assay. Acyclovir (ACV; 10 *μ*g/mL) was included as a positive control. The graph represents data from 2 independent experiments (ANOVA, Bonferroni's Multiple Comparison Test; ^∗^ *P* < 0.0001). (c) TCID_50_ obtained from Vero cells preincubated for 24 h with Transferon (20 *μ*g/mL) or medium prior to HSV-1 infection (Student's* t* test). (d) Titration of samples obtained from Vero cells infected 24 h with HSV-1 and simultaneously treated with medium (open circles) or 20 *μ*g/mL of Transferon (closed circles). ACV (10 *μ*g/mL; triangles) was included as a positive control. Sum-of-squares* F* test showed no differences between the TCID_50_ from medium- and Transferon-treated cells (*P* = 0.7527). The graph represents data from 2 independent experiments.

We measured the antiviral activity of Transferon by (i) preincubating HSV-1 with the drug before adding it to the target (Vero) cells (Figure 4(a)) and (ii) simultaneously incubating target cells with HSV-1 and various Transferon concentrations (10 pg/mL–10 *μ*g/mL) (Figure 4(b)). Transferon did not show direct antiviral effect on either experimental system. Preincubation of target cells with Transferon for 24 h before the viral challenge did not prevent HSV-1 infection (Figure 4(c)), suggesting that Transferon does not modify the target cell-susceptibility to infection. Additionally, we evaluated the effect of Transferon on virus replication. The titration of HSV-1 on Transferon-treated samples showed that the TCID_50_ is not different from that of control samples. Values obtained from nonlinear regression fit were −log_10_ = 2.711 versus −log_10_ = 2.752, respectively (Figure 4(d)). These results, obtained by a colorimetric assay, correlate with the visual analysis of CPE (Supplemental Figure 1 available online at http://dx.doi.org/10.1155/2014/146305), as reported for others viruses [28], and demonstrate that Transferon has no effect on HSV-1 replication.

### 3.5. Effects of Transferon on Systemic Cytokines

**Figure 5:**
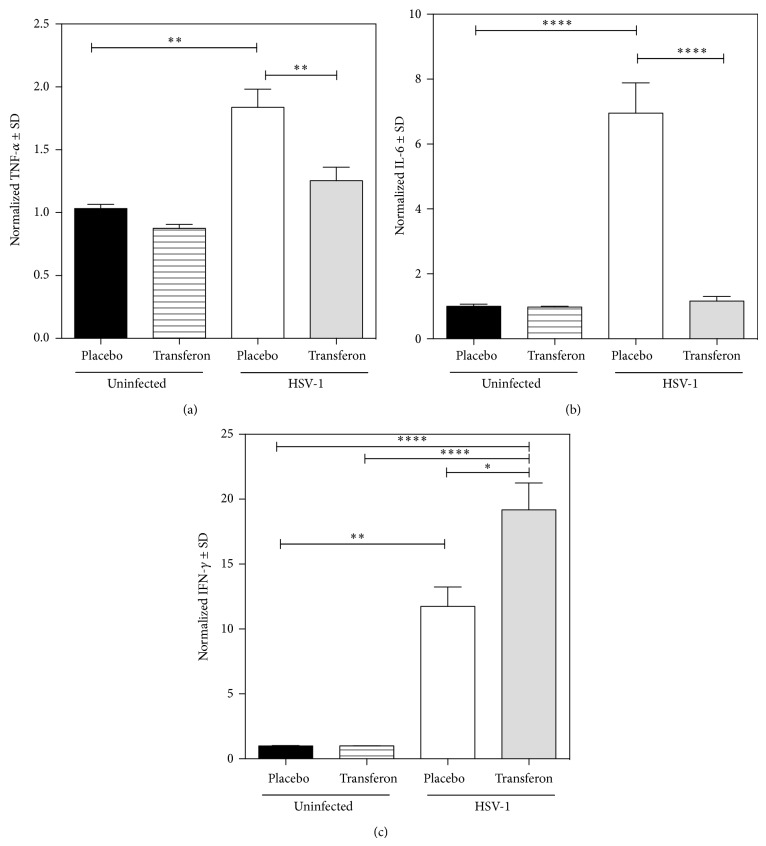
Quantitative analysis of blood (serum) cytokine levels. Transferon (Batch 12C04; 0.75 *μ*g/mouse) significantly reduced the concentrations of TNF-*α* (a) and IL-6 (b) at day 9 postinfection, but it increased that of IFN-*γ* at day 7 (c). Transferon administration had no effect on uninfected mice. All measurements were normalized to placebo-treated, uninfected controls. The graphs represent data of 2 independent experiments (ANOVA, Bonferroni's Multiple Comparison Test; ^∗^ *P* < 0.05; ^∗∗^ *P* < 0.01; ^∗∗∗^ *P* < 0.001; ^∗∗∗∗^ *P* < 0.0001).

Blood (serum) cytokine levels were measured at days 7 and 9, before the period of high mortality. TNF-*α*, IL-6, and IFN-*γ* levels rose in HSV-1-infected and placebo-treated mice versus uninfected mice (Figures 5(a)–5(c)). In contrast, Transferon did not change the cytokines levels in uninfected mice. Treatment of HSV-1-infected mice with Transferon significantly decreased TNF-*α* and IL-6 levels at day 9, compared with placebo-treated animals (Figures 5(a) and 5(b). In contrast, Transferon further increased IFN-*γ* levels at day 7 (Figure 5(c)).

## 4. Discussion

All 27 batches of Transferon in this study complied with quality standards with regard to their attributes. As shown for batches that were produced in 2010-2011 [8], we noted high homogeneity in microbial content, peptide concentration, molecular weight, and time of retention of the main peaks by UPLC. These results demonstrate that is possible to produce a mixture of peptides that have been extracted from complex raw materials, such as lysed human leukocytes.

To evaluate the activity of these batches, a murine model of herpes was standardized and validated. Transferon partially protected animals from the HSV-1-induced weight loss, which correlated with greater survival. This model was chosen because DLEs are effective in clinical studies of herpetic infections. DLEs significantly reduce the average duration of the acute phase and the frequency of recurrences in herpetic infections as successfully as antiviral drugs [15, 16]. All batches of Transferon significantly increased survival dose-independently, indicating that it is biologically active over a wide range of doses (10-fold). Thus, our murine model of herpes can be used to measure efficacy—a fundamental attribute of biological drugs—in DLEs.

However, the herpes murine model has several disadvantages. The variability that is intrinsic to a whole-animal model is significant; such a model requires the use of many animals. Also, more time is needed to evaluate activity than for an* in vitro* experiment.

Transferon has no direct virucidal or antireplicative effects on HSV-1 nor cytoprotective effects on target cells, suggesting that the* in vivo* activity of Transferon is mediated by its effects on the immune system. We found that HSV-1-infected mice had higher serum concentrations of TNF-*α*, IL-6, and IFN-*γ* compared with uninfected controls. Consistent with these findings, in astrocyte cultures, HSV-1 infection upregulates TNF-*α*, IL-6, and NF-*κ*B in a Toll-like receptor (TLR)-3-dependent manner [31]. Administration of Transferon to HSV-1-infected mice downregulates TNF-*α* and IL-6, suggesting that it suppresses innate immune responses. The modulation of proinflammatory cytokines by DLEs has been associated with reduced inflammation-associated tissue damage by pathogens [32, 33].

Conversely, Transferon increased IFN-*γ*, a cytokine that is produced during the adaptive phase of immunity, suggesting that Transferon indirectly stimulates the activation of T lymphocytes that are specific for HSV-1. IFN-*γ* effects resistance against HSV-1 infection [34], and its upregulation in DLE-treated herpes patients favors a positive clinical response [17] and limits relapses [16]. Although the mechanisms of action of DLEs have not been determined completely, our results indicate that they have differential effects on innate and adaptive immunity.

## 5. Conclusions

Our analysis of 27 batches of Transferon demonstrate that (i) the cutaneous model of herpes can be used to evaluate the biological activity of DLEs, such as Transferon; (ii) the assay can be used as a routine test for batch release; (iii) Transferon is produced with high homogeneity between batches, meeting the standards that are required for hemoderivatives that are intended for clinical use; (iv) Transferon does not have direct virucidal, cytoprotective, or antireplicative effects; and (v) the protective effects of Transferon in our murine model correlate with changes in serum cytokine levels.

## Supplementary Material

Supplemental Figure 1: Titration HSV-1 by cytopathic effect evaluation (120 h). Samples of Vero cells infected with HSV-1 (2000 PFU) in absence (medium) or presence of Transferon (24 h) were collected and titrated. Aciclovir (ACV) was used as positive control.

## Figures and Tables

**Table 1:** Quality attributes of evaluated batches of Transferon.

Batch	Protein (mg/mL)	MAB^#^ (CFU/mL)	FF&Y^&^ (CFU/mL)	Endotoxin (EU/mL)
12A01	0.392	<1	<1	<0.05
12C01	0.438	<1	<1	0.197
12C03	0.432	<1	<1	<0.05
12C04	0.445	<1	<1	<0.05
12C05	0.419	<1	<1	0.167
12C06	0.394	<1	<1	<0.125
12C07	0.428	<1	<1	<0.125
12D08	0.427	<1	<1	<0.05
12F09	0.427	4	<1	<0.05
12G11	0.347	<1	<1	<0.05
12H14	0.421	<1	<1	0.098
12H15	0.406	<1	<1	<0.05
12K20	0.430	<1	<1	<0.125
12K21	0.436	<1	<1	<0.125
12L22	0.432	<1	<1	<0.125
12L23	0.448	<1	<1	<0.125
13A03	0.417	<1	<1	<0.125
13B07	0.423	<1	<1	<0.25
13C08	0.408	<1	<1	<0.25
12M24	0.397	<1	<1	0.125
12M25	0.422	<1	<1	<0.125
13A02	0.425	<1	<1	<0.125
13C09	0.445	<1	<1	<0.25
13A04	0.398	<1	<1	1.0
13C10	0.397	<1	<1	<0.25
13B05	0.389	<1	<1	<0.25
13B06	0.389	<1	<1	<0.25

^#^Mesophilic aerobic bacteria; ^&^Filamentous fungus and yeast.
