# Supplementary figures and images for: Herpes Murine Model as a Biological Assay to Test Dialyzable Leukocyte Extracts Activity
Source: J Immunol Res. 2015 Apr 23;2015:146305. doi: 10.1155/2015/146305 (PMC4423021; doi:10.1155/2015/146305)

-log Dilution Factor

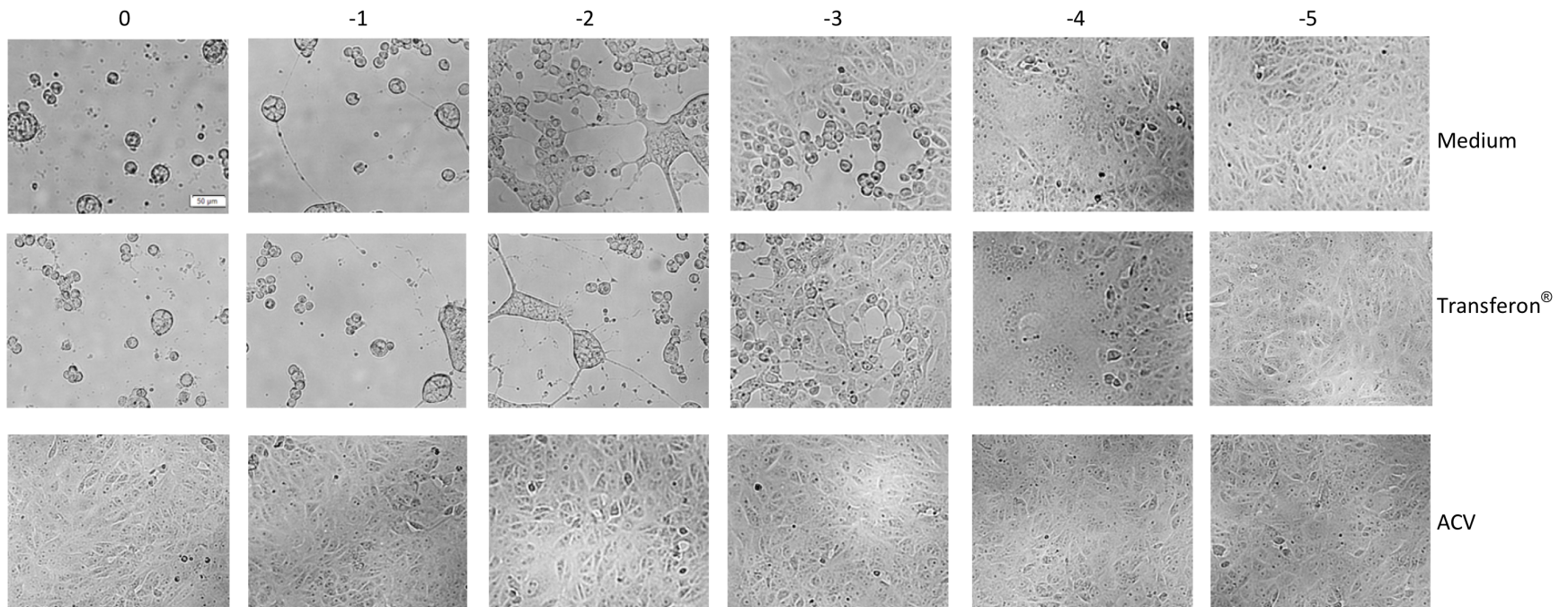

Supplemental Figure 1

Supplement: Supplementary file 1 — Supplemental Figure 1: Titration HSV-1 by cytopathic effect evaluation (120 h). Samples of Vero cells infected with HSV-1 (2000 PFU) in absence (medium) or presence of Transferon (24 h) were collected and titrated. Aciclovir (ACV) was used as positive control. [file 146305.f1.pdf]
